# Species-Specific Pathogens Drive Distinct Structural and Inter-Kingdom Dynamics in the Mulberry Leaf Microbiome

**DOI:** 10.3390/microorganisms14081724

**Published:** 2026-08-06

**Authors:** Quan Chen, Chenxi Yang, Wen Yang, Jiequn Ren, Zhangyun Zheng, Mingan Pan, Feng Huang

**Affiliations:** 1 Chongqing Three Gorges Academy of Agricultural Sciences, Wanzhou 404155, China; chenquan0616@126.com (Q.C.);; 2 Plant Protection Research Institute, Guangdong Academy of Agricultural Sciences, Key Laboratory of Green Prevention and Control on Fruits and Vegetables in South China Ministry of Agriculture and Rural Affairs, Guangdong Provincial Key Laboratory of High Technology for Plant Protection, Guangzhou 510640, China

**Keywords:** mulberry, leaf microbiome, ITS, 16S rRNA, mulberry brown spot

## Abstract

A plant pathogen is a deterministic factor affecting the plant microbiome. However, whether the influence of different pathogen species on the plant microbiome is consistent is not well-known. In this study, mulberry leaves with similar spots caused by two diseases—mulberry brown spot (MBS, caused by *Neophloeospora maculans*) and mulberry snags rotten leaves disease (MSRL, caused by *Boeremia exigua* var. *mori*)—along with asymptomatic leaves, were collected. Their endophytic fungal and bacterial communities were sequenced. The occurrence of MSRL reduced leaf fungal richness and diversity (observed species from 329 to 203; Shannon diversity index from 3.6 to 2.6, *p* < 0.05; phylogenetic diversity index from 45.8 to 20.6, *p* < 0.05), along with leaf bacterial diversity; in contrast, the occurrence of MBS had little effect on both fungal and bacterial richness and diversity. Both diseases significantly altered the composition and structure of leaf fungal communities, whereas only MBS did so for bacterial communities. For the dominant genera, the fungal genera, except *Cercospora*, were differently affected by the occurrence of MBS and MSRL, while the bacterial genera *Pseudomonas* (from 8.3% to 30.6%, *p* < 0.05 for MBS; from 23.1% to 28.1% for MSRL), *Massilia* (0.2% to 7.7%, *p* < 0.05; 2.1% to 6.1%), *Chryseobacterium* (0.1% to 0.3%; 2.7% to 7%), *Pantoea* (2.6% to 3.7%; 0.4% to 1%), and *Aureimonas* (0.7% to 1.4%; 1.3% to 1.7%) were consistently enriched by both diseases. In addition, *Chryseobacterium* (LDA score = 4.6, *p* < 0.01) was detected as a biomarker after the occurrence of MSRL. Intriguingly, there were four times more significant fungi–bacteria positive associations induced by the occurrence of MSRL compared to MBS (10.2% to 2.5%). Taken together, the responses of mulberry leaf microbiome to pathogen infection vary in response to the pathogen species; although several bacterial genera were consistently enriched, whether their enrichment reflects their antagonistic roles in fungal—bacterial inter-kingdom interactions remains to be determined and should be interpreted with further experimental tests.

## 1. Introduction

Mulberry (*Morus alba* L.) is a woody, flowering, and deciduous perennial plant [1]. It is widely distributed in many countries in Asia, Europe, and America. In China, it has a very long cultivation and usage history [1]. For example, it is well-known that mulberry leaves have been used to feed silkworms, silkworms produce silk which is woven into the famous Chinese silk. In addition, mulberry is also a documented traditional Chinese herb; its leaves are efficient in removing body wind heat and fever, moistening human liver and lungs, and alleviating obesity [2]. Until now, mulberry leaves are frequently found with new excellent nutritional values and outstanding functional properties, suggesting their great potential in the process of functional foods and nutraceuticals [3,4].

Based on the information above, the most of the functions of mulberry are from its leaves, which are highly sensitive to various fungal diseases [5]. The brown spot, powdery mildew, and red rust are the most prevalent mulberry leaf diseases; their occurrence and outbreak seriously impairs leaf growth, leading to lower leaf yields and deteriorated leaf quality [6,7,8]. In China, these fungal diseases are commonly seen in different mulberry production areas, such as Guangxi, Zhejiang, and Jiangxi [5]. For example, Chen et al. (2022) found at least 10 common plant diseases in mulberry in a single Province of Jiangxi [5]. Leaf spots were the most common symptoms that can be induced by multiple fungal species, including *Neophloeospora* sp., *Colletotrichum* sp., *Fusarium* sp., *Phoma* sp., and *Boeremia* sp. [5]. A single needle-like spot can ruin the economic value of an entire mulberry leaf, yet the application of fungicides is strictly restricted, because these leaves are likely to be used to prepare tea and other food products [5,7]. This dilemma implies that finding alternative disease control methods is urgent for the production of mulberry.

Studies on plant–pathogen–microbiome tripartite interaction suggest that the fungal and bacterial species from the plant microbiome are promising in controlling plant pathogens [9,10]. For example, Bai et al. (2026) constructed high-diversity synthetic bacterial communities that significantly reduced the root rot caused by *Fusarium oxysporum* in *Astragalus* plants [11]. Liu et al. (2026) constructed synthetic microbial communities from the rhizosphere microbiomes of *Fusarium* wilt-resistant banana varieties, which, upon inoculation, reduced *Fusarium* wilt pathogen abundance to approximately 37.7% [12]. Pathogen infection causes plant cell death, the plant cell nutrients are released to feed the pathogen and plant microbiome [13,14]. In feedback, the plant microbiome response to pathogen infection through direct interaction and nutrient competition, which usually leads to the enrichment of specific fungal and bacterial species, along with the lowering of the richness and diversity of the whole microbial community [15,16]. The enriched fungal and bacterial species may include members of interest for disease control, as their prevalence is largely attributable to their competitive advantage over the pathogen for plant cell-released nutrients [11,12]. However, the response of the plant microbiome to pathogen infection in most plant species remains poorly understood, hindering the development of synthetic microbial consortia for disease control.

Mulberry brown spot (MBS) and mulberry snags rotten leaves disease (MSRL) are two of the most devastating fungal diseases on mulberry in China [17,18,19]. MBS can be dated back to the 1980s. Since then, the disease has been reported in many different countries, including India, Japan, Turkey, Korea, Poland, the USA, and Brazil [20,21]. In comparison, MSRL has only been locally reported in Guangxi Zhuang Autonomous Region, Chongqing Municipality, and Jiangxi Province, China since the year of 2011 [5,17,22]. Both diseases can produce brown spots on mulberry leaves, and their outbreak poses a huge risk to the mulberry industry [18,22]. For example, it was estimated that over 50% of mulberry orchards were affected by MBS in Yunnan Province each year, and the incidence of diseased plants varied from 58.9% to 100% [23]. In addition, the first discovery of MSRL indicated that this disease was highly prevalent in the mulberry production areas of West Guangxi, and the incidence of diseased plants was up to 90% in infected orchards [22]. In this study, the mulberry leaves with either MBS or MSRL and their asymptomatic leaves were collected and used for fungal ITS and bacterial 16S amplicon sequencing. To provide insights into developing phyllosphere microbiomes to combat leaf spot diseases, the response patterns of mulberry endophytic leaf microbiome to different fungal pathogen species were compared in: (1) the fungal and bacterial richness and diversity, (2) the composition and structure of fungal and bacterial communities, (3) the relative abundances of major fungal and bacterial taxa, and (4) the fungi–bacteria association.

## 2. Methods and Materials

### 2.1. Plant Leaf Sampling

**Figure 1:**
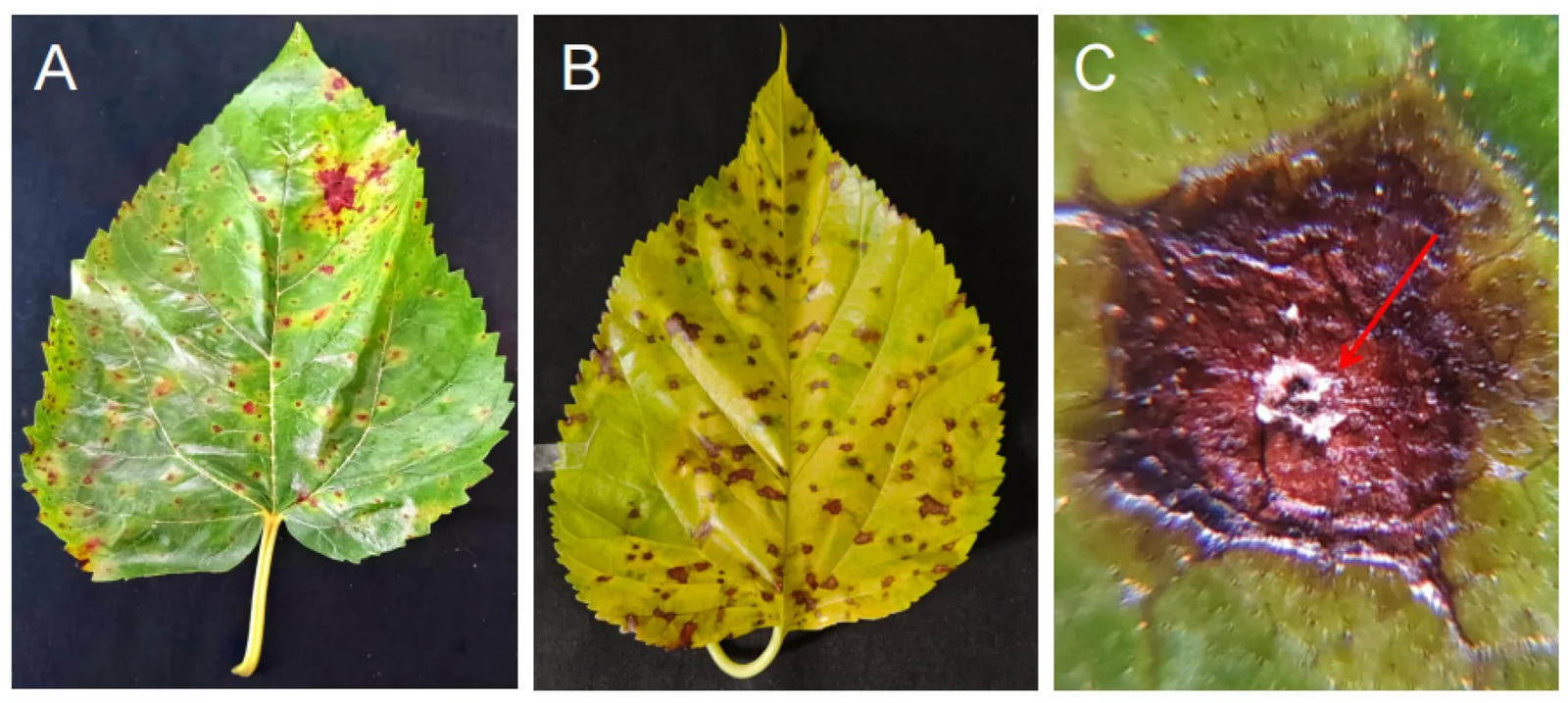

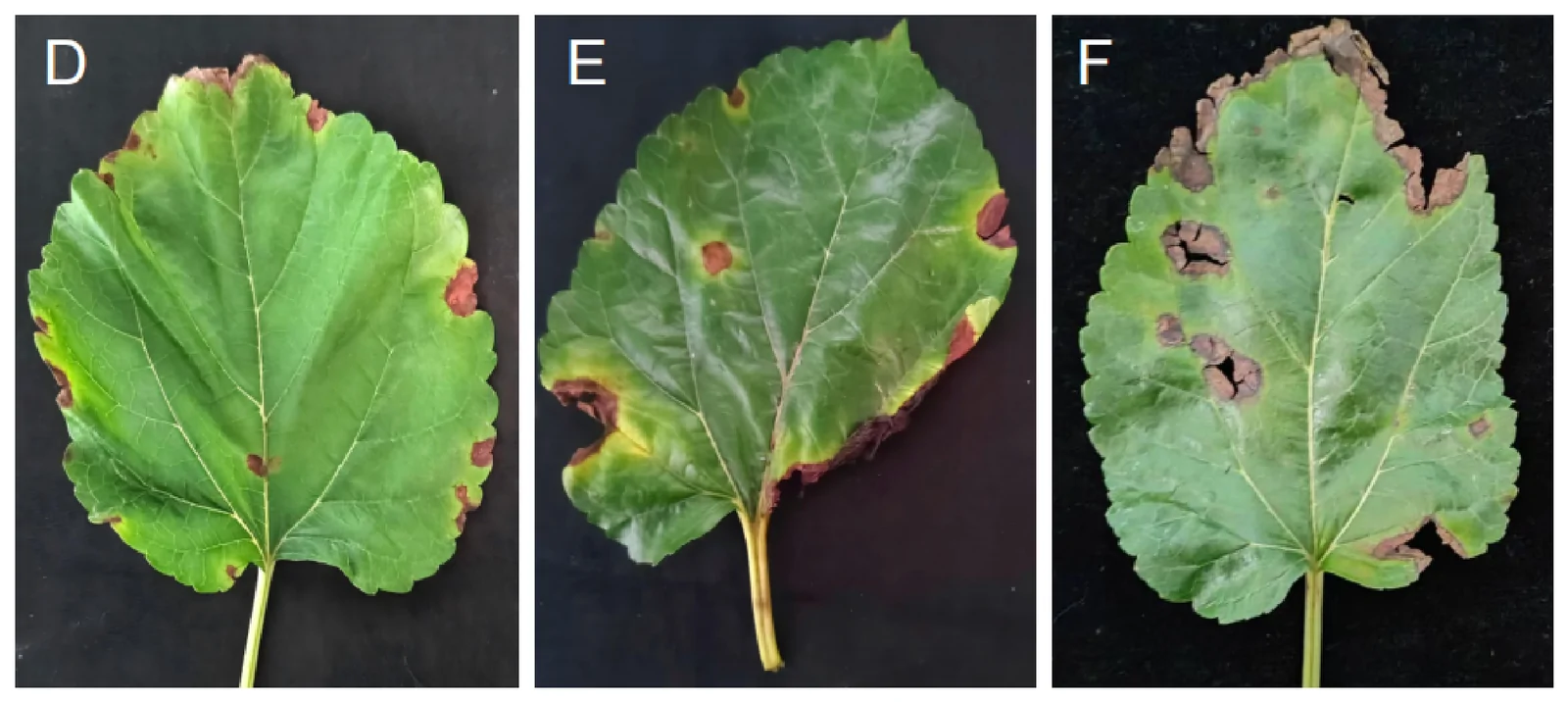
MBS and MSRL leaf symptoms. Leaf spots caused by mulberry brown spot (MBS, (**A**–**C**)) and mulberry snags rotten leaves disease (MSRL, (**D**–**F**)). The red arrow in C indicates the powdery mass formed in the late stage of a brown pot.

On 12 May 2025, two mulberry orchards, Shengkong and Xintian, in Baishi, Qianjiang, Chongqing, China, were selected for leaf sampling. The orchard Shengkong has broken out with mulberry brown spot (MBS), while the other orchard has broken out with mulberry snags rotten leaves disease (MSRL). The samples of MBS and MSRL were collected from the two orchards, respectively. Both diseases can produce red to brown spots on mulberry leaves which are hard to distinguish by symptoms at an early stage (Figure 1). For a diseased tree, a total of six leaves with spots were collected; all the spots whose diameter varied from 2 to 10 mm were cut out and pooled as a sample of either MBS or MSRL. For control, healthy trees were selected without any visible disease symptoms and insect bites, and then six asymptomatic leaves (Asym) were cut from each tree. The leaves were cut into pieces about the same size as leaf spots, and all the leaf pieces from the same tree were pooled as a sample. The leaves were surface-sterilized by soaking in 75% ethanol for 15 s, 2% bleach for 15 s, and then being rinsed in sterile water for three times [24]. The leaf spots were confirmed through tissue isolation of potential fungal pathogens, the appeared fungal isolates were compared to our lab-stored fungal strains of *Neophloeospora maculans* and *Boeremia exigua* [17]. All the samples were ground with liquid nitrogen and stored under −80 °C for further use.

### 2.2. Leaf Microbial DNA Extraction and Amplicon Sequencing

The microbial genomic DNA was extracted from 1 g leaf powder per sample using the CTAB (cetyltrimethylammonium bromide) method [25]. Partial sequences of fungal nuclear ribosomal internal transcribed spacer 1 (ITS1 rDNA) and bacterial 16S rRNA V3 + V4 region were amplified using polymerase chain reaction (PCR) with two primer pairs of ITS1F (5′-CTTGGTCATTTAGAGGAAGTAA-3′)/ITS2-2043R (5′-TGCTGCGTTCTTCATCGATGC-3′) and 335F (5′-CADACTCCTACGGGAGGC-3′)/769R (5′-ATCCTGTTTGMTMCCCVCRC-3′), respectively [26,27]. The PCR was carried out using the Phusion High-Fidelity PCR Master Mix (New England Biolabs Inc., Ipswich, MA, USA); the reaction cycle was set: 94 °C for 5 min, followed by 35 cycles of 94 °C for 45 s, 56 °C for 30 s, 72 °C for 30 s, and a final extension at 72 °C for 10 min. Each PCR was conducted in triplicate, and then the PCR products of the same sample were pooled for library preparation. PCR products were verified on 2% agarose electrophoresis gel, and target DNA were collected and cleaned with Qiagen Gel Extraction Kit (Qiagen, Hilden, Germany). Negative extraction and PCR controls were processed in parallel, and their lack of amplification on agarose gels confirmed the absence of detectable contamination. Sequencing libraries were prepared using TruSeq^®^ DNA PCR-Free Library Preparation Kit (Illumina, San Diego, CA, USA), the quality of libraries were assessed using Qubit@ 2.0 Fluorometer (Thermo Scientific, Sunnyvale, CA, USA) and Agilent Bioanalyzer 2100 system (Agilent Technologies, Santa Clara, CA, USA). The sequencing was carried out on Illumina NovaSeq 6000 platform (Illumina, San Diego, CA, USA) by BioMarker Co., Ltd. (BioMarker, Beijing, China).

### 2.3. Bioinformatics Analysis

A total of 22 samples were sequenced, comprising nine samples of MBS, three of asymptomatic controls for MBS, seven of MSRL, and three of asymptomatic controls for MSRL. After sequencing, 3,460,617 fungal and 3,519,689 bacterial paired-end raw reads were generated from 22 leaf samples. The raw reads were filtered using Trimmomatic version 0.39 [28] and cutadapt v1.9.1 [29] following the following workflow: (1) remove adapters, primers, and poly bases; (2) discard short sequences (<200 bp) and sequences with detected number < 3; (3) discard sequences with more than 10% unknown nucleotides and less than 80% Q-value > 20 bases. After filtering, 2,998,415 (136,292 in average, varied from 116,144 to 142,798) fungal and 2,967,547 (134,889, 120,884 to 141,220) bacterial clean reads were generated. Clean reads were assembled using FLASH (v1.2.7, http://ccb.jhu.edu/software/FLASH/ accessed on 21 November 2025) and input into DADA2 for de-noising and removal of chimeric sequences on the QIIME2 2020.6 platform [30,31]. The reads were clustered to amplicon sequence variants (ASVs) in DADA2; representative sequences of fungal ASVs (fASVs) and bacterial ASVs (bASVs) were annotated by blasting to the UNITE (https://unite.ut.ee/ accessed on 27 November 2025) and SILVA138 (http://www.arb-silva.de/ accessed on 27 November 2025) database, respectively.

### 2.4. Statistical Analysis

The ASVs tables of the leaf fungal and bacterial communities, combining ASVs numbers and taxa assignments, were generated in QIIME2 2020.6 [31]. The total number of ASVs of each sample was normalized to the sample with the least ASVs number (114,211 for fungi, 85,934 for bacteria); then the numbers of observed species, Shannon, Simpson, ACE, Chao1, Good’s coverage, phylogenetic diversity, and the relative abundances of each taxa level were calculated based on the normalized ASVs table. Further analysis were performed in R platform (v. 4.3.1) [32]. The homogeneity of multivariate dispersion between healthy and diseased samples was assessed using PERMDISP with 999 permutations based on Bray–Curtis distance matrices (*p* > 0.05). Subsequently, PERMANOVA was applied to the same distance matrices to test for differences in fungal and bacterial community composition and structure between groups, using the vegan package (v. 2.6-8) [33]. Differences in composition and structure were analyzed using principal coordinate analysis (PCoA) in the Ape package (v. 5.8-1) [34], and then the first two PCoAs were plotted using ggplot2 (v. 4.0.3) [35]. Core. ASVs included the ASVs presented in at least one third of the total samples. Linear discriminant analysis effect size (LEfSe) analysis was conducted using the microeco package [36]. Fungi–bacteria correlation was analyzed using the Hmisc package (https://hbiostat.org/R/Hmisc/ accessed on 23 March 2026) and displayed in a heatmap using the pheatmap package (v. 1.0.13) [37]. Wilcoxon test and Kruskal−Wallis test were used for the comparison of two and multiple groups, respectively. The FDR adjusted *p* values lower than 0.05 were accepted as significant except in correlation analysis (*p* < 0.01).

## 3. Results

### 3.1. The Occurrence of MBS and MSRL in Leaf Fungal and Bacterial Communities

**Figure 2:**
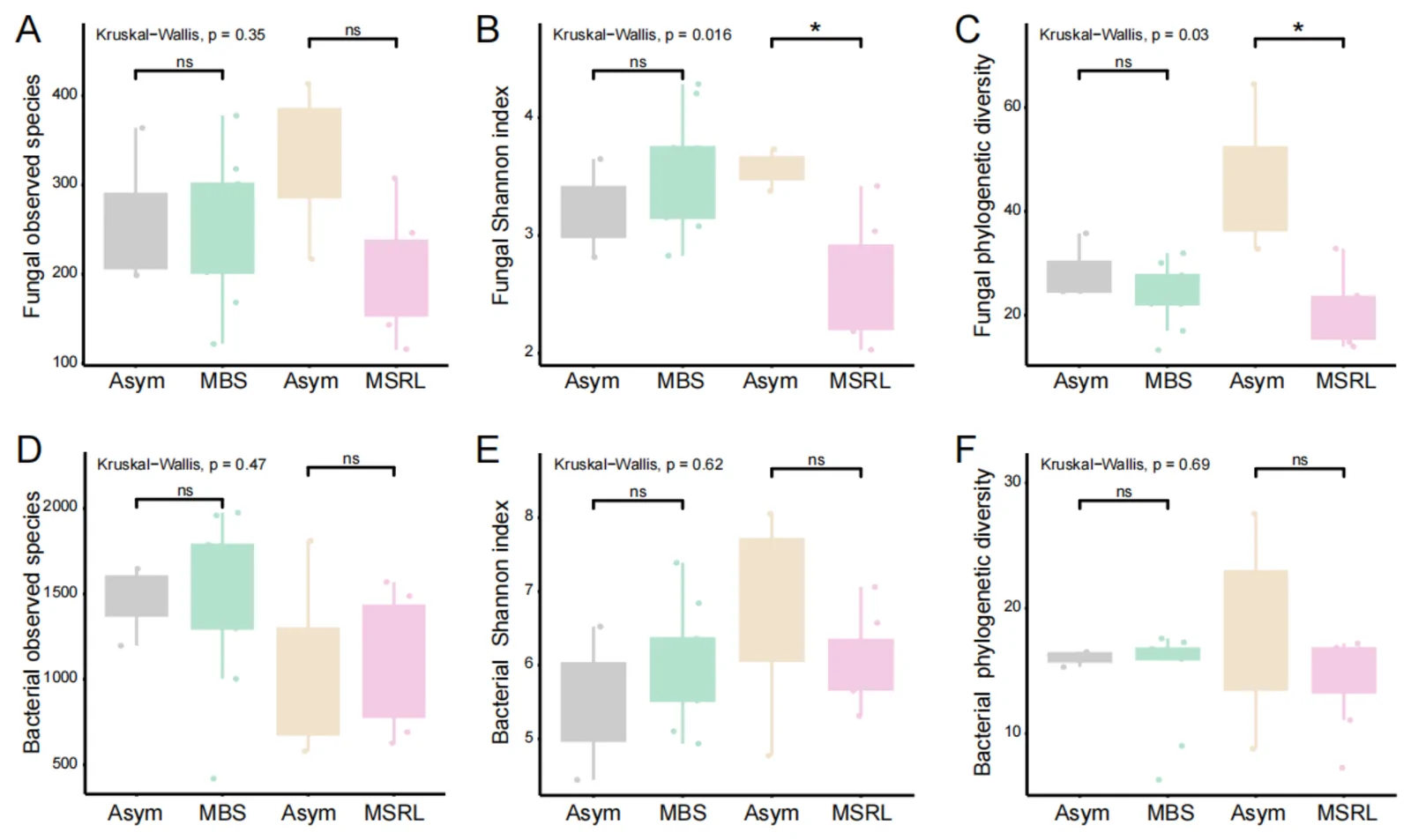
Alpha diversity. Fungal and bacterial richness and diversity of mulberry brown spot, mulberry snags rotten leaves disease, and their asymptomatic (Asym) leaves. (**A**–**C**), the number of fungal observed species, Shannon index, and phylogenetic diversity; (**D**–**F**), the number of bacterial observed species, Shannon index, and phylogenetic diversity. “*” indicates *p* < 0.05 by Wilcoxon test, “ns” indicates not significant.

The occurrence of MBS did not significantly alter the observed species, Shannon diversity index, and phylogenetic diversity of both the fungal (Figure 2A–C) and bacterial (Figure 2D–F) communities. In contrast, the occurrence of MSRL consistently reduced the observed species, Shannon diversity index, and phylogenetic diversity of the fungal, but not of the bacterial, communities. Specifically, the occurrence of MSRL reduced the fungal observed species from 329 of the asymptomatic leaves (Asym) to 203 of the MSRL leaves (Figure 2A), the Shannon diversity index from 3.6 to 2.6 (*p* < 0.05, Figure 2B), and the phylogenetic diversity from 45.8 to 20.6 (*p* < 0.05, Figure 2C).

**Figure 3:**
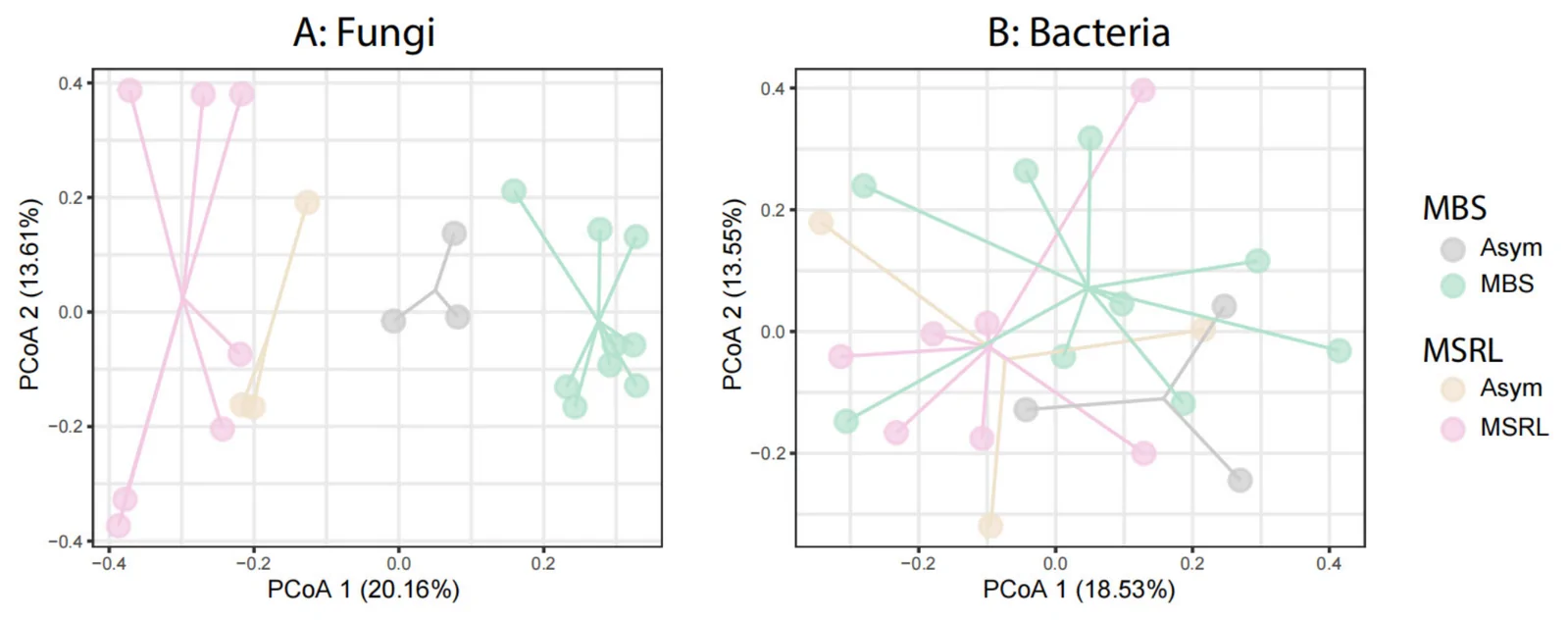
Microbial beta diversity. Principal coordinate analysis (PCoA) of the fungal (**A**) and bacterial (**B**) communities of mulberry brown spot, mulberry snags rotten leaves disease, and their asymptomatic leaves.

In addition, the occurrence of both diseases significantly altered the composition and structure of leaf fungal communities (*p* < 0.001, Figure 3A). The composition and structure of the fungal communities of Asym leaves of MBS and MSRL were significantly different (*p* < 0.001); they were further altered by MBS and MSRL in opposite directions along the PCoA 1 (20.2% variation explained, Figure 3A). The occurrence of MBS, but not of MSRL, significantly altered the composition and structure of leaf bacterial communities (*p* < 0.001, Figure 3B). The variation between Asym and MBS leaves was explained through PCoA 2 (13.6% variation explained, Figure 3B).

### 3.2. The Occurrence of MBS and MSRL on Dominant Fungal and Bacterial Taxa

**Figure 4:**
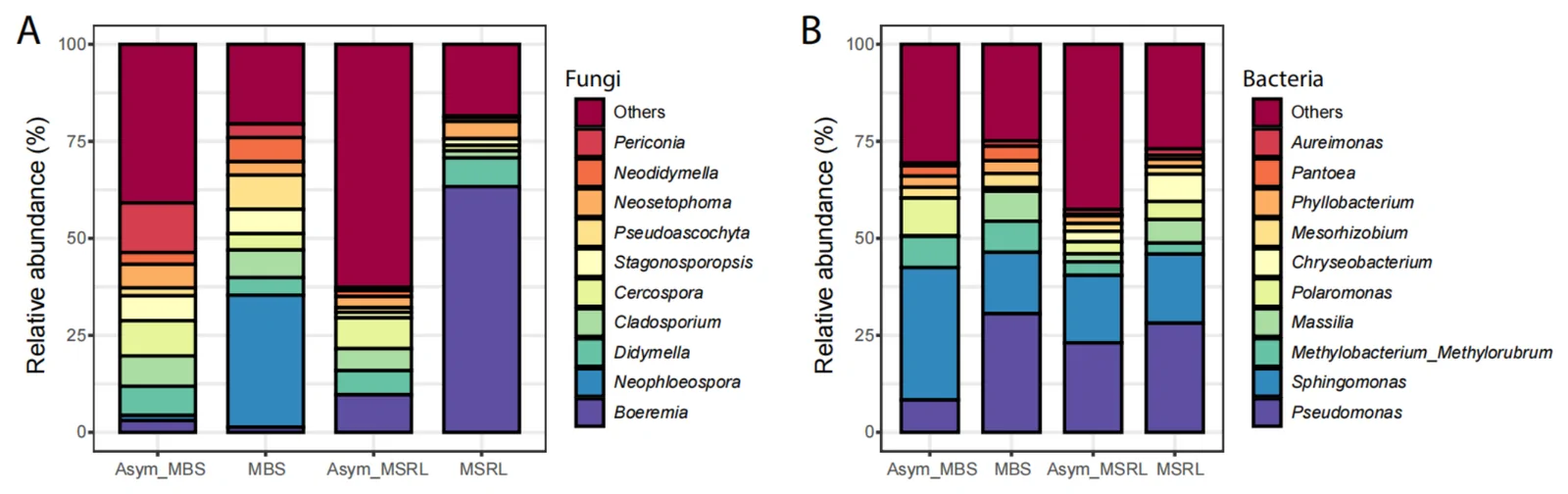
Microbial top genera. The relative abundances of dominant fungal (**A**) and bacterial (**B**) genera of mulberry brown spot, mulberry snags rotten leaves disease, and their asymptomatic leaves.

At the genus level, the endophytic microbiome of mulberry leaves was dominated by the fungal genera of *Boeremia*, *Neophloeospora*, *Didymella*, *Cladosporium*, *Cercospora*, *Stagonosporopsis*, *Pseudoascochyta*, *Neosetophoma*, *Neodidymella*, *Periconia* (Figure 4A), and the bacterial genera of *Pseudomonas*, *Sphingomonas*, *Methylobacterium*_*Methylorubrum*, *Massilia*, *Polaromonas*, *Chryseobacterium*, *Mesorhizobium*, *Phyllobacterium*, *Pantoea*, and *Aureimonas* (Figure 4B). For the fungal genera, the occurrence of MBS increased the relative abundances of *Neophloeospora* (from 1.3% in the asymptomatic leaves to 34% in the diseased leaves, *p* < 0.01), *Pseudoascochyta* (from 2.1% to 8.8%), and *Neodidymella* (from 3.1% to 6.2%) and reduced the relative abundances of *Boeremia* (from 3% to 1.3%), *Didymella* (from 7.5% to 4.5%), *Cercospora* (from 9.1% to 4.2%), *Neosetophoma* (from 6.1% to 3.5%), and *Periconia* (from 12.8% to 3.5%, *p* < 0.05). While the occurrence of MSRL increased the relative abundances of *Boeremia* (from 9.7% to 63.3%, *p* < 0.01), *Didymella* (from 6.2% to 7.4%) and *Neosetophoma* (from 2.9% to 4.4%), it reduced the relative abundances of *Cladosporium* (from 5.7% to 1.8%, *p* < 0.05), *Cercospora* (from 7.9% to 1.4%, *p* < 0.05), *Pseudoascochyta* (from 1.2% to 0.1%), and *Neodidymella* (from 1.5% to 1%).

For the bacterial genera, the occurrence of MBS increased the relative abundances of *Pseudomonas* (from 8.3% to 30.6%, *p* < 0.05), *Massilia* (from 0.2% to 7.7%, *p* < 0.05), *Chryseobacterium* (from 0.1% to 0.3%), *Mesorhizobium* (from 2.7% to 3.6%), *Phyllobacterium* (from 2.9% to 3.3%), *Pantoea* (from 2.6% to 3.7%), and *Aureimonas* (from 0.7% to 1.4%) and reduced the relative abundances of *Sphingomonas* (from 34.1 to 15.8%, *p* < 0.05) and *Polaromonas* (from 9.9% to 0.7%, *p* < 0.05). While the occurrence of MSRL increased the relative abundances of *Pseudomonas* (from 23.1% to 28.1%), *Massilia* (2.1% to 6.1%), *Polaromonas* (from 3.1% to 4.6%), *Chryseobacterium* (from 2.7% to 7%), *Pantoea* (from 0.4% to 1%) and *Aureimonas* (from 1.3% to 1.7%), it reduced the relative abundances of *Methylobacterium*_*Methylorubrum* (from 3.5% to 2.9%).

### 3.3. Indicative Fungal and Bacterial Taxa of MBS and MSRL

**Figure 5:**
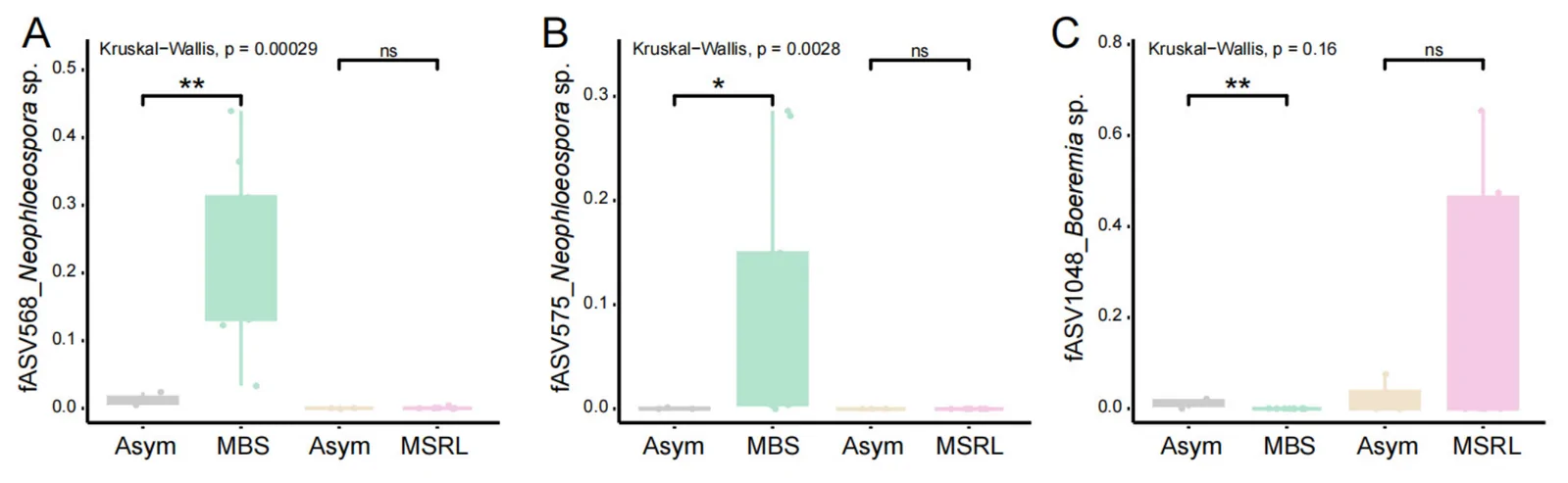
Pathogen phylotypes. The relative abundances of the pathogen phylotypes. (**A**,**B**) The phylotypes of the pathogen of mulberry brown spot, *Neophloeospora maculans*; (**C**) the phylotypes of the pathogen of mulberry snags rotten leaves disease, *Boeremia exigua*. “*” indicates *p* < 0.05, “**” indicates *p* < 0.01, “ns” indicates not significant by Wilcoxon test.

The substantial increase in *Neophloeospora* (26.2 times, hosting the causal agent of MBS) in the MBS leaves was represented by two fungal ASVs of fASV568 (Figure 5A) and fASV575 (Figure 5B). The relative abundance of fASV568 increased from 1.3% in the asymptomatic leaves to 24.2% in the diseased leaves (*p* < 0.01) and that of the fASV575 increased from 0 to 9.3% (*p* < 0.05). The increase in *Boeremia* (6.5 times, hosting the causal agent of MSRL) in the MSRL leaves was represented by the fASV1048, whose relative abundance increased from 2.5% in the asymptomatic leaves to 22.6% in the diseased leaves (Figure 5C). Interestingly, the fASV1048 significantly decreased from 1.2% in the asymptomatic leaves to 0 in the MBS-diseased leaves (*p* < 0.01, Figure 5C).

**Figure 6:**
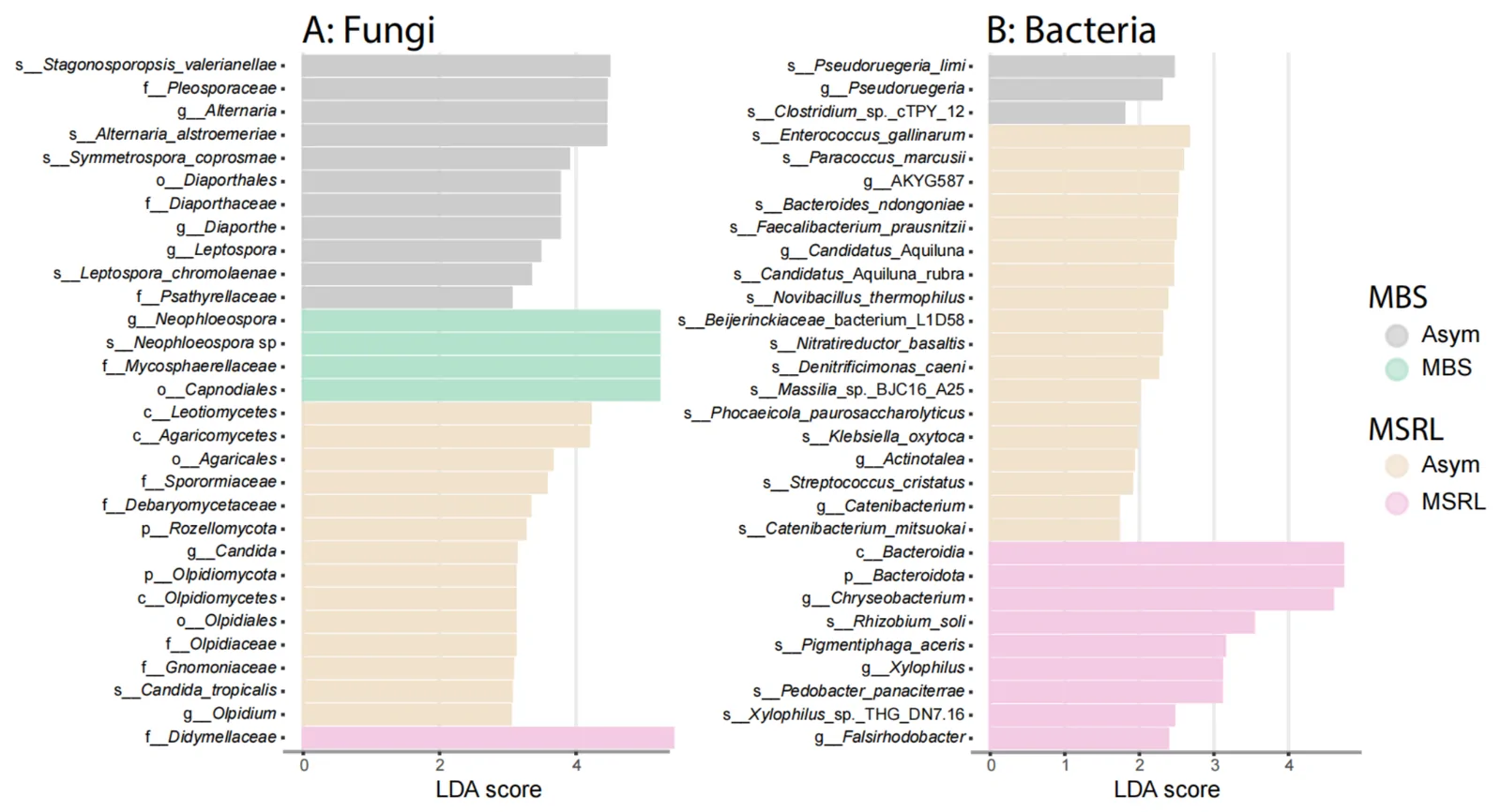
Disease biomarkers. Fungal (**A**) and bacterial (**B**) biomarkers of mulberry brown spot, mulberry snags rotten leaves disease, and their asymptomatic leaves by linear discriminant analysis effect size (LEfSe) analysis.

In the biomarker analysis, the Asym leaves of MBS were typically occupied by two fungal species of *Alternaria alstroemeriae* (LDA score = 4.4, *p* < 0.01) and *Stagonosporopsis valerianellae* (LDA score = 4.5, *p* < 0.01), a fungal genus of *Alternaria* (LDA score = 4.4, *p* < 0.01), and a fungal family of *Pleosporaceae* (LDA score = 4.4, *p* < 0.01, Figure 6A), while the Asym leaves of MSRL were typically occupied by two fungal classes of *Agaricomycetes* (LDA score = 4.2, *p* < 0.01) and *Leotiomycetes* (LDA score = 4.2, *p* < 0.01, Figure 6A). After the occurrence of MBS, their leaf microbial communities were occupied by the pathogen-related fungal taxa, including *Neophloeospora* sp. (LDA score = 5.2, *p* < 0.01), and its upper taxa *Neophloeospora* (LDA score = 5.2, *p* < 0.01), *Mycosphaerellaceae* (LDA score = 5.2, *p* < 0.01), and *Capnodiales* (LDA score = 5.2, *p* < 0.01, Figure 6A). Similarly, after the occurrence of MSRL, their leaf microbial communities were occupied by the pathogen located fungal family of *Didymellaceae* (LDA score = 5.4, *p* < 0.01, Figure 6A), along with a bacterial genus of *Chryseobacterium* (LDA score = 4.6, *p* < 0.01) and its upper taxa Bacteroidia (LDA score = 4.7, *p* < 0.01), *Bacteroidota* (LDA score = 4.7, *p* < 0.01, Figure 6B).

### 3.4. The Occurrence of MBS and MSRL on the Fungi–Bacteria Co-Occurrence Pattern

**Figure 7:**
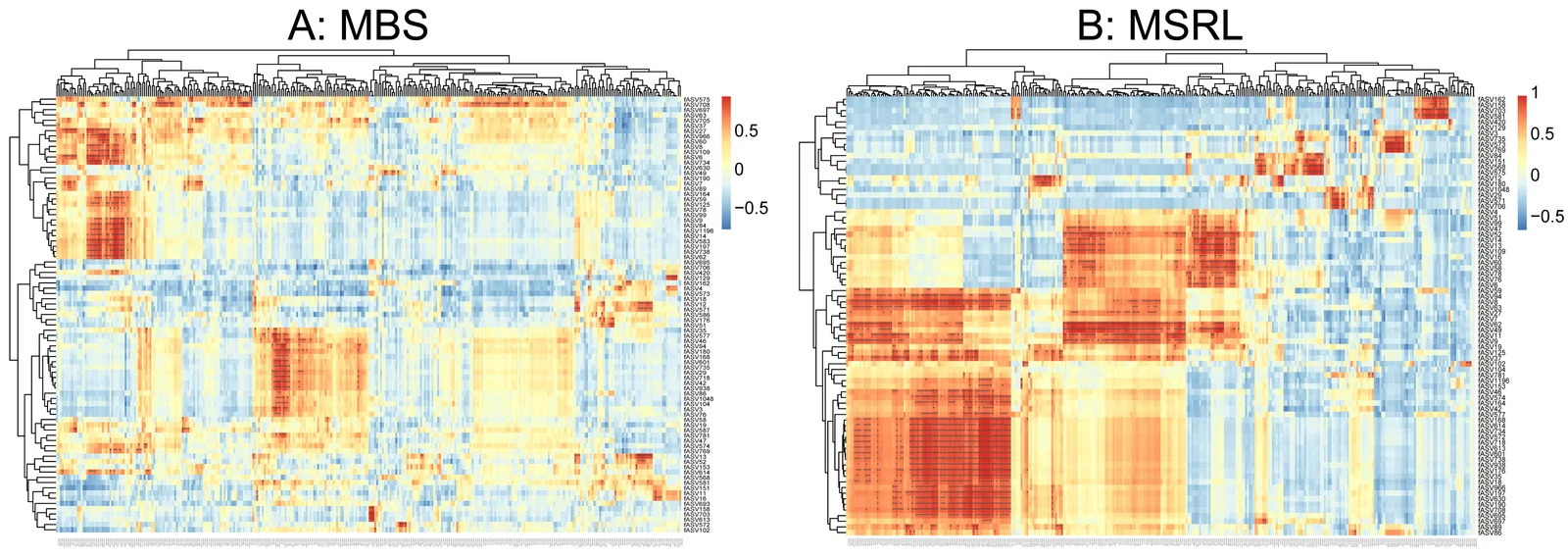
Fungi–bacteria correlation. Correlation between fungal and bacterial core ASVs. (**A**) Mulberry brown spot; (**B**) mulberry snags rotten leaves disease. The grid color represents the correlation coefficients, “*” denotes *p* < 0.01.

The 83 fungal ASVs (fASVs) and 328 bacterial ASVs (bASVs) produced 27,224 pairs of fungi–bacteria correlations in the microbial communities of MBS leaves. However, five fASVs were excluded due to their extremely low relative abundances (close to 0), thus 25,584 pairs of fungi–bacteria correlations in the microbial communities of MSRL leaves remained. For MBS leaves, 12,443 (45.7%) positive (*R*^2^ > 0) and 14,780 (54.3%) negative (*R*^2^ < 0) fungi–bacteria correlations were detected—687 (2.5%) positive correlations were significant (*p* < 0.01), and no negative correlation was significant (Figure 7A)—while for MSRL leaves, 14,623 (57.2%) positive and 10,805 (42.2%) negative fungi–bacteria correlations were detected—2622 (10.2%) positive correlations were significant (*p* < 0.01), and no negative correlation was significant (Figure 7B).

## 4. Discussion

Mulberry brown spot (MBS), caused by *Neophloeospora maculans*, produces brown or tan-brown spots, later covered with white to slightly red powdery masses on leaves (Figure 1A–C). Mulberry snags rotten leaves disease (MSRL), caused by *Boeremia exigua* var. *mori*, produces grayish-brown spots, which eventually dry out, crack, and form shot holes on leaves (Figure 1D–F). At an early stage, both fungal diseases cause similar brown spots on mulberry leaves that are difficult to distinguish by symptoms alone [17,19]. In this study, leaf samples showing spots of MBS and MSRL were collected at an early stage. The spot diameter ranged from 2 to 10 mm, and no symptoms of drying or shot holes were observed. The spots were cut out and the leaves from the same tree were mixed to form a sample. In total, nine and seven leaf spot samples were collected, but only three asymptomatic leaf samples were collected for MBS and MSRL, respectively. During the outbreak period of MBS and MSRL, nearly the whole mulberry orchards were affected, which made it hard to find mulberry trees without any visible symptoms [22,23]. For this reason, only three asymptomatic leaf samples were collected for each disease (MBS and MSRL).

Even though causing obvious leaf cell necrosis (Figure 1A–C), the occurrence of MBS did not significantly affected leaf fungal and bacterial richness and diversity. In comparison, the occurrence of MSRL significantly reduced leaf fungal diversity, and showed some negative influences on leaf fungal richness and bacterial diversity. From previous studies, the occurrence of plant diseases, like MSRL, usually has negative influences on the richness and diversity of plant microbiome [11,16]. This may be caused by: (1) the transformation of diverse plant microbial communities to pathogen-dominated microbial communities, and (2) the acceleration of microbial competition by plant cell necrosis and nutrients release after pathogen infection [14,16]. From our study, it is observed that the fungal communities of MBS and MSRL were transformed to *Neophloeospora*- and *Boeremia*- dominated communities, respectively (Figure 4A). But it seems that the growth of *Neophloeospora* has little influences on the richness and diversity of both the leaf fungal and bacterial communities. Compared to *B. exigua* var. *mori*, *N. maculans* is a slow-growing fungus; it grew about 1.1 mm per day on PDA medium under 25 °C, while the former one grew 5.7 to 8.6 mm per day under the same conditions [18,22]. In addition, the symptom development of *N. maculans* needed five to seven days, longer than the two to four days required for *B. exigua* var. *mori* following the same inoculation method [19,22]. These findings may suggest that *N. maculans* is a relatively mild fungus in terms of nutrient competition and microbial co-occurrence, as its growth space is largely shared by other opportunistic microbes. However, there are few studies available on the virulence, infection mechanism, and microbial co-existence of these two fungal pathogens, which restricts our further understanding of their different influences on the mulberry leaf microbiome.

In addition, the occurrence of MBS and MSRL significantly altered the composition and structure of mulberry leaf fungal communities (Figure 3A) but in different directions. As we mentioned above, the leaf fungal communities were transformed to pathogen-dominated communities after disease occurrence. Specifically, the relative abundances of *Neophloeospora* and *Boeremia* were increased by 26.2 (from 1.3% to 34%) and 6.5 (from 9.7% to 63.3%) times from asymptomatic leaves to MBS and MSRL leaves, their increase may account for a large part of the variation in the composition and structure of the fungal communities. Furthermore, the increase in *Neophloeospora* and *Boeremia* affected the relative abundances of other dominant fungal genera (except *Cercospora*) in opposite directions. This pattern can explain why the variations in the composition and structure of the mulberry leaf fungal communities caused by MBS and MSRL were also in opposite directions. But why the increase in *Neophloeospora* and *Boeremia* exerted opposite influences on other major fungal genera remains unclear. Further studies on fungal cooperation and competition at the strain level are still needed.

Furthermore, only the occurrence of MBS—not MSRL—significantly altered the composition and structure of mulberry leaf bacterial communities (Figure 3A). In the asymptomatic leaves of MSRL, the relative abundance of *Boeremia* was 9.7%, which was still the most abundant fungal genera in their fungal communities. This result implies that *B. exigua* var. *mori* is a latent pathogen, it colonizes and amplifies in mulberry leaves before the spot appears [38,39]. Accordingly, the latent *B. exigua* var. *mori* may interact with the bacterial communities of asymptomatic leaves, an interaction that attenuates the compositional and structural variation between MSRL and asymptomatic leaves [40,41]. In comparison, the occurrence of MSRL exerted more pervasive and uniform effects on both the major bacterial genera (Figure 4B) and the fungi–bacteria co-occurrence pattern (Figure 7). This observation may also explain why MSRL only moderately reshapes the composition and structure of the mulberry leaf bacterial community.

Fungi–bacteria associations play a crucial role in plant defense against diseases. For example, bacteria enriched in plants affected by Citrus melanose and Astragalus root rot effectively inhibited the growth and symptom development of their respective fungal pathogens, *Diaporthe citri* and *Fusarium oxysporum* [42,43]. In this study, abundant positive fungi–bacteria associations were detected in both MBS and MSRL leaves. However, such positive microbial correlations may reflect mutualism, spatial co-occurrence, or shared nutritional preferences, rather than genuine ecological interactions [24,44]. Therefore, their actual role in suppressing fungal pathogens requires experimental validation. Interestingly, no significant negative fungi–bacteria associations were detected in either MBS or MSRL leaves. In fungal pathogen-suppressive microbial communities, positive associations often predominate over negative ones [44,45], which may account for the absence of significant negative correlations in our data. Alternatively, the lack of significant negative fungi–bacteria associations may be attributed to the limited number of healthy leaf samples, which may have compromised the statistical power to detect such interactions.

Even though with different effects on mulberry leaf bacterial communities, both diseases consistently enriched the bacterial genera *Pseudomonas*, *Massilia*, *Chryseobacterium*, *Pantoea*, and *Aureimonas*. The same set of bacteria may function antagonistically against both the fungal pathogens *N. maculans* and *B. exigua* var. *mori*, raising the possibility of incorporating them into synthetic communities for dual disease biocontrol [24,46]. For example, culture-dependent studies have shown that mulberry endophytes—including isolates of *Pantoea agglomerans*, *Bacillus subtilis*, and *Burkholderia cepacia*—can confer protection against diverse fungal diseases [47,48,49]. Notably, the genus *Chryseobacterium* was identified as a biomarker for MSRL, suggesting its potential role in disease suppression. Many *Chryseobacterium* species are known to promote plant growth and health [50,51]. For instance, a rice-derived strain produces volatile organic compounds that inhibit *Magnaporthe oryzae* (>80%) and induce systemic resistance [51]. Collectively, our findings highlight pathogen species as a key determinant of microbiome response patterns and identify a core set of bacteria with potential for biocontrol applications. The bacteria enriched by MBS and MSRL may possess analogous functions and therefore merit isolation and functional investigation.

## 5. Conclusions

Although mulberry brown spot (MBS) and mulberry snags rotten leaves disease (MSRL) both cause similar brown spots on mulberry leaves, they differentially reshape the leaf microbiome. Specifically: MSRL, unlike MBS, reduces fungal richness, as well as both fungal and bacterial diversity. Both diseases alter the composition and structure of fungal communities, each enriched by its respective pathogenic genus—*Neophloeospora* for MBS and *Boeremia* for MSRL. MBS primarily shapes bacterial community composition and structure, whereas MSRL exerts broader and more uniform effects on major bacterial genera and fungi–bacteria associations. Notably, both diseases consistently enrich the same five bacterial genera: *Pseudomonas*, *Massilia*, *Chryseobacterium*, *Pantoea*, and *Aureimonas*. We tentatively hypothesize that the five shared enriched genera may represent general stress indicators or biocontrol candidates, though we acknowledge that this conclusion is drawn from limited data and should therefore be treated with due caution.

## Data Availability

The raw data supporting the conclusions of this article will be made available by the authors on request.
